# Comparative Effectiveness of Antidepressant Medication versus Psychological Intervention on Depression Symptoms in Women with Infertility and Sexual Dysfunction

**DOI:** 10.22074/ijfs.2018.5229

**Published:** 2018-01-15

**Authors:** Hajar Pasha, Zahra Basirat, Mahbobeh Faramarzi, Farzan Kheirkhah

**Affiliations:** 1 Infertility and Reproductive Health Research Center, Health Research Institute, Babol University of Medical Sciences, Babol, Iran; 2 Departments of Psychiatry, Faculty of Medicine, Babol University of Medical Sciences, Babol, Iran

**Keywords:** Bupropion, Depression, Infertility, Psychotherapy, Sexual Dysfunction

## Abstract

**Background:**

Fertility loss is considered as a challenging experience. This study was conducted to compare the effectiveness of antidepressant medication and psychological intervention on depression symptoms in women with infertility and sexual dysfunctions (SD).

**Materials and Methods:**

This randomized, controlled clinical trial study was completed from December 2014 to June 2015 in Babol, Iran. Of the 485 participants, 93 were randomly assigned in a 1:1:1 ratio to psychosexual therapy (PST), bupropion extended-release (BUP ER) at a dose of 150 mg/d, and control (no intervention) groups. The Beck Depression Inventory (BDI) was completed at the beginning and end of the study. Duration of study was eight weeks. Statistical analyses were performed by using paired-test and analysis of covariance.

**Results:**

The mean depression score on the BDI was 22.35 ± 8.70 in all participants. Mean BDI score decreased significantly in both treatment groups (PST: P<0.0001, BUP: P<0.002) from baseline to end of the study, whereas intra-individual changes in BDI score were not significant in the control group. The decrease in mean BDI score was greater with PST compared to BUP treatment (P<0.005) and the control group (P<0.0001). The PST group showed greater improvement in depression levels (severe to moderate, moderate to mild) in comparison with the two other groups (P<0.001). Drug treatment was well tolerated by the participants in the BUP group.

**Conclusion:**

PST can be a reliable alternative to BUP ER for relieving depression symptoms in an Iranian population of women with infertility and SD (Registration number: IRCT2015042721955N2).

## Introduction

Infertility is an emotionally challenging experience in women’s lives. Fertility loss can cause various mental problems such as feeling of loss of control, low self- esteem, stress, depression, marital distress, and sexual dissatisfaction (1-5). The prevalence of depression ranges between 40-50% among women with infertility (1, 6). Also, sexual dysfunction (SD) is a common problem in women with infertility, where the rate is 46.6% in a sample of Iranian infertile women (7). Moreover, there is an association between SD and depression; namely, a review of the literature reveals that depression has been frequently associated with sexual impairments (8, 9). 

Therefore, depression itself may contribute to SD and vice-versa (4, 10, 11). The studies showed that the depression leads to decreasing in success rate with *in vitro* fertilization (IVF). In addition, depression found to have an inversely correlation with pregnancy. The higher levels of depression is associated with lower rate of pregnancy (12). 

Both cognitive behavioral therapy and drug therapy are effective in treating depression in women with infertility (3). A review of the literature revealed that psychosocial interventions had a beneficial effect on depression and the well-being of women with infertility (13). Psychotherapy or sex therapy had antidepressant effects and improved sexual function (3, 14, 15). For drug therapy, bupropion is used to treat depression related to sexual dysfunction. It is a norepinephrine and dopamine reuptake inhibitor (16), and is effective in the improvement of depression (17). Bupropion is an effective antidepressant and can be used as a supplemental treatment to reverse antidepressant-induced SD (18). Although one study showed that there was not a significant difference in depression rating scale between the bupropion users with placebo groups (19). 

While behavioral and pharmacological treatments are effective in treating depression and SD in infertility, few studies have assessed these treatments for depression in infertile women with sexual dysfunction. To our knowledge, there are no published studies that have compared the effect of psychosexual therapy (PST) and bupropion, in the extended release (ER) formulation, in this population. Hence, the present study aimed to compare the effectiveness of bupropion extended-release versus psychological intervention on depression symptoms in depressed women with infertility and SD in Fatemeh Zahra Infertility and Reproductive Health Research Center of Babol University of Medical Sciences, Iran.

## Materials and Methods

 The authors carried out an open-label randomized controlled clinical trial between December 2014 to June 2015 at the Fatemeh Zahra Infertility and Reproductive Health Research Center, Babol, Iran. The study design was confirmed by the Ethics Committee (4930, 12 Jan 2014) and then registered in IRCT. Primary objective of this project was based on the treatment of SD with subsequent improvement of depression symptoms.

Women with infertility less than 45 years of age were eligible for the research under the following criteria: a score of =10 in beck depression index (BDI), a score of =26.55 on Iranian version of the female sexual function index (IV-FSFI), an infertility duration of greater than one year, were literate, ability to read and write, weren’t undergone any fertility treatment in the next 2 months and were sexually active in the past four weeks. Subjects were excluded from the study if they had a history of seizures, were taking medications that could lower the seizure threshold or were known to effect sexual function, had a history of head trauma, had a major change in living conditions, or had psychological support. Exclusion criteria also included serious medical conditions and mental health problems under the treatment of a physician, having actively suicidal, having major depressive disorder (MDD) in the clinical interview by a female psychologist.

**Fig.1:**
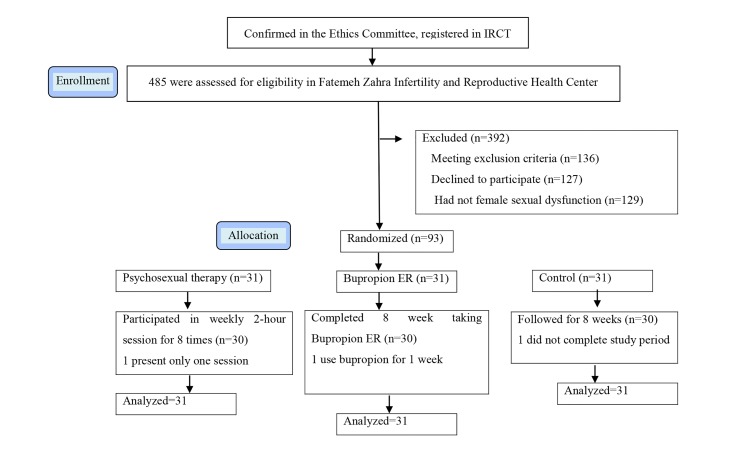
Flow diagram of participants through each stage of a randomized clinical trial.

Subjects have been assessed for eligibility by two midwives with no clinical involvement in the study. Sample size was calculated in accordance with 22 subjects in each group, with accuracy=6.6, confidence interval (CI)=95%, and approximate SD=6.8 based on previous studies (3, 17, 20, 21) and power=90% for each group. After consideration of the corrected sample size formula (n'=√Kn, k=2) (22), a total of 93 eligible infertile women were selected through computer-aid randomization in equal 3 groups (31 person in each group). A total of 93 depressed women with infertility and SD were randomly allotted in a 1:1:1 ratio to three equal groups as follows: i. PST, ii. Pharmacotherapy (BUP), and iii. Control (Fig .1). In this study both the researcher and the participants were not blinded. 

The PST interventions focused on educational programs that mainly contained eight weeks of two-hour sessions including mindfulness-based cognitive therapy (MBCT), relaxation training, behavior sex therapy (Mixed method) based on the Crowe and Ridley model, and also booklet of tranquility, mindfulness and medication (23-25) in the shape of group discussions, questions and answers, lectures, booklets, and CD with groups of 9-13 members. The pharmacotherapy group was treated with bupropion ER at a dose of 150 mg/d (Wellban Extended release, Abidi Company, Iran) for up to eight weeks.

The control group did not receive intervention, but educational package was given to them, and were referred to a sex therapy clinic after the end of study. Study duration, and bupropion dosage were different in previous studies (4- 24 week, and 100-450 mg/day) (3, 17, 18, 26, 27). Therefore, intervention period, and dosing for bupropion in this study was considered 8 week, and 150 mg orally once a day in patients with depression symptoms treated for sexual dysfunction. Adverse events (AEs), vital sign, and Anthropometric measurements (weight, height) were serially conducted for each of groups by researcher, were summarized at each data collection schedule assessment point. During these visits, the previous bag of bupropion was collected and new bags of bupropion were given to be used for the next two weeks. Participants were also contacted by phone biweekly to monitor for any complaint, or any change in their health status(for any reason). At the same time the researcher could be contacted by phone at any time.

A total of 90 participants completed the study. At first, the research protocol was described for participants, and then written informed consent was obtained from each subject. A secure and confidential environment was considered for collecting data. The researcher used a binder for each participant to keep individual information.

Demographic information was collected. All subjects completed the BDI at baseline and after treatment at the end of the study. The BDI is a self-reported measure consisting of 21 questions to assess the severity of depression symptoms, Created by Beck in 1961, it has an approved validity (0.89); reliability (0.96) during the first decade following its introduction. The translated and Persian of BDI had Cronbachs alpha 0.87 in the Iranian population.

The intensity of the item rates on a 4-point scale (0-3) and the test is scored by summing the ratings given to each of the 21 items. The total score range between 0 and 63 and the results range as follows: 0-9 as no depression, 10-18 as mild depression, 19-29 as moderate depression, and 30 and greater as severe depression (28-30). A score =10 in BDI was considered ‘‘at risk’’ for depression (3). The FSFI, used to measure sexual dysfunction. It contains 19 items in six different subscales of sexual desire, arousal, lubrication, orgasm, satisfaction, and pain. A score of =26.55 indicates sexual dysfunction. The validity and reliability of the Iranian version of FSFI is high (Cronbach’s alpha 0.70-0.9) (4, 7).

## Statistical analysis

Data analyses were performed using Paired t tests, Pearson’s correlation, χ^2^ test, analysis of covariance (ANCOVA), and Tukey’s test (SPSS software, version 21) in an ITT analysis, with P<0.05 indicating statistical significance. Paired t tests was applied to show significant inter individual changes in BDI score within each treatment group. The chi-square test was used for comparing categorical variables between three groups. The ANCOVA was used as a statistical technique to control for variability (with baseline BDI scores as a covariate variable).

Tukey’s test was used for pair wise comparisons. Subsequent tests included the homogeneity of variances, the linear relationship between the dependent variable and the covariate, and the normality of distributions (Skewness and Kurtosis test). The change of depression level from baseline to end of study was calculated for each group. Improvement in depression symptoms was defined as a pre- to post treatment decrease in BDI depression level (severe to moderate, moderate to mild, and mild to no depression). Worsening of depression was defined as an increase in BDI depression level from baseline to end of study (mild to moderate, moderate to severe).

## Results

The demographic characteristics of subjects are showed in Table 1. The majority of participants were unemployed (78.5%), while the majority of the participants’ spouses were self-employed (44.1%). The mean age of participants was 29 ± 5.44 years. The economical situation in more than one third of participants was poor (38.7%). The type of housing for the majority of participants was private (65.6%). Nearly two thirds (62.4%) of participants had primary infertility. There were no statistically significant differences in baseline factors, occupation, husband occupation, educational level, economic status, type of housing, infertility type, and infertility etiology between the three groups (Table 1).

The mean BDI score of participants was 22.35 ± 8.70 at baseline. Mean BDI scores at baseline and at the end of study for the three groups are given in Table 2. Paired t tests showed significant inter individual changes in BDI score within each treatment group (PST, P<0.0001, BUP, P<0.002, Table 3). Depression symptoms decreased significantly in both PST and BUP groups from baseline to end of study. The intra-individual changes were not significant in control group (P=0.105).

**Fig.2:**
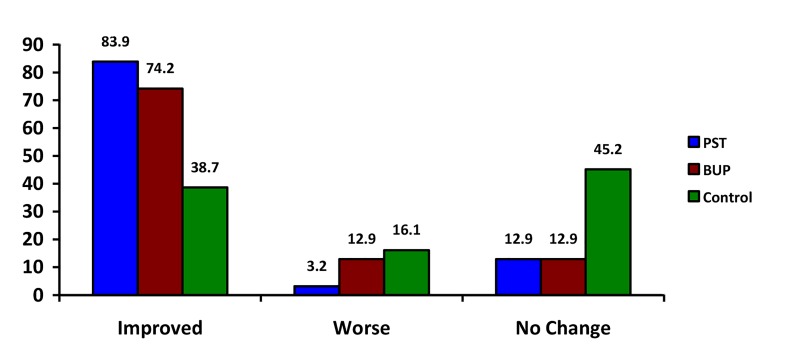
The changes of final levels of baseline depression in three groups. PST; Psychosexual therapy, BUP ER; Bupropion extended-release. n=93 (each group of 31 participants). P<0.001 (χ^2^ test).

Changes in depression level (pre to post treatment) showed that 79.1% of participants in the treatment groups(PST and BUP) improved from their baseline depression level, while 8.05% had a worse depression level, and 12.9% had no change. In the control group, 38.7% of participants improved from their baseline depression level, while 16.1% had a worse depression level, and 45.2% had no change. The improved depression levels were showed more in PST group compared to others groups (Fig .2).

**Table 1:** Distribution of the participarts according to the sociodemographic charecteristics


Variable	Treatment group		Control n=31 n (%)	P value	Total n=93 n (%)
	PST n=31 n (%)	BUPER n=31 n (%)			

Occupation				0.168	
Unemployed	21 (67.7)	27 (87.1)	25 (80.6)		73 (78.5)
Employed	10 (32.3)	4 (12.9)	6 (19.4)		20 (21.5)
Husband occupation				0.810	
Unemployed	1 (3.2)	1 (3.2)	1 (3.2)		3 (3.2)
Worker	8 (25.8)	10 (32.3)	5 (16.1)		23 (24.7)
Employee	7 (22.6)	8 (25.8)	11 (35.5)		26 (28.0)
Self-employed	15 (48.4)	12 (38.7)	14 (45.2)		41 (44.1)
Type of housing				0.716	
Private	20 (64.5)	19 (61.3)	22 (71.0)		61 (65.6)
Rental	11 (35.5)	12 (38.7)	9 (29.0)		32 (34.4)
Infertility type				0.242	
Primary	17 (54.8)	23 (74.2)	18 (58.1)		58 (62.4)
Secondary	14 (45.2)	8 (25.8)	13 (41.9)		35 (37.6)
Education				0.183	
0-12 (Y)	24 (77.4)	25 (80.6)	19 (61.3)		68 (73.1)
>12 (Y)	7 (22.6)	6 (19.4)	12 (38.7)		25 (26.9)
Infertility etiology				0.806	
Female	2 (6.5)	2 (6.5)	5 (16.1)		9 (9.7)
Male	11 (35.5)	11 (35.5)	9 (29)		31 (33.3)
Both	9 (29)	10 (32.2)	7 (22.6)		26 (28)
Unknown	9 (29)	8 (25.8)	10 (32.3)		27 (29)


PST; Psychosexual therapy and BUPER; Bupropion extended-release. χ2 test was used for comparing categorical variables between three groups.

The change from baseline in BDI score was analyzed by ANCOVA and significant differences between the three groups were found (P<0.001). Pair wise comparisons on mean BDI showed that only PST decreased significantly compared to control group (P<0.0001). The decrease in mean BDI score in the BUP group was not significantly different compared to control group (P<0.282). There was a significant diference in mean BDI scores between PST and BUP groups (P<0.005) (post-hoc ANCOVA). Statistical power for this analysis was approximately than 95.4%. As a result, the assumption of research on the effects of therapeutic interventions on BDI score with a probability of 95.4% in infertile women was accepted (Table 2). There was statistically significant negative relationships between the mean of FSFI score with BDI score (P<0.001). Bupropion is well tolerated.

**Table 2:** Covariance analysis test for total score in beck depression and pair wise comparisons in the groups


Variable	Sum of squares	Mean square	df	F statistics	Observed power	P value

Depression	2114.060	2114.060	1	24.149	0.998	0.0001
Group	1427.404	713.702	2	8.153	0.954	0.001


BDI; Beck depression inventory, PST; Psychosexual therapy, BUP ER; Bupropion extended-release, n=93 (each group of 31 participants), df; Degrees of freedom, and F; Test statistic. ANCOVA test was used to compare the change from baseline in BDI score between the three groups; post-hoc ANCOVA was used for pair wise comparisons. There was a significant diference in mean BDI scores between PST and control groups (P<0.0001), PST and BUP ER groups (P<0.005); but not between BUP and control groups (P<0.282).

**Table 3:** The mean scores of BDI in three groups of infertile women at beginning and end of the study


Variable BDI	Pre-test Mean ± SD	Post-test Mean ± SD	P value

PST	24.59 ± 7.76	10.42 ± 9.01	0.0001
BUP ER	22.42 ± 10.70	16.09 ± 11.81	0.002
Control	20.06 ± 6.83	17.35 ± 10.46	0.105


BDI; Beck depression inventory, PST; Psychosexual therapy, BUP ER; Bupropion extended-release, n=93 (each group of 31 participants). Paired t test was used to compare the pre-to-post depression BDI mean score in each group.

## Discussion

Study participants with SD showed where they were at risk for depression symptoms and had a high average of depression scores. This finding suggests that women with infertility and SD are more likely to experience symptoms of depression. Previous studies by Pasha et al. (1), Pakpour et al. (4) and Peyvandi et al. (31) show similar results, where female SD put women with infertility at high risk for depression. Sexual problems are severely distressing experiences, and may be important factors in the development of depression (7). Consistent with this, marital dissatisfaction is associated with an increase in severe depression (31).

We found that BDI scores decreased significantly from baseline at the end of study in each treatment group (PST and BUP). In both treatment groups combined, more than two thirds of participants showed improvement in their depressive symptoms levels (pre to post treatment). The similar studies showed that both psychosocial and pharmaceutical therapeutic strategies, such as psychotherapy and antidepressants, are well established in the treatment of depression (3, 17). A review of psychological interventions by Boivin (14) reported that psychotherapy decreased negative affect in infertile people. Psychotherapy or sex therapy had a beneficial effect on the management of psychological symptoms affecting sexual function in women (3, 15).

Also bupropion is an effective antidepressant medication, which is used to treat remission of depressive disorder. The effectiveness of bupropion in improving depression found in clinical trials with the drug (27, 32). Furthermore, this research indicated a statistically significant negative relationship between sexual function and depression symptoms at patients treated for sexual dysfunction. In line with finding, another studies found that higher scores of FSFI are correlated with lower depression scores (21). There was a negative correlation between depression with sexual function and marital satisfaction (11). It was associated with lower depression, higher marital satisfaction reverse antidepressant- induced SD (21, 27, 33). Therefore, it is important to note that the resolution of depression scores can be related to relieving sexual problem in depressed patients (8, 14, 18, 34). This is because the SD and depression are interchangeable, so that the treatment of one will change the others. 

After adjusting for baseline values, data showed a significant improvement in depression symptoms for women exposed to the psychosexual intervention compared with women in the BUP and control groups. Group PST was better than bupropion treatment in improving depression symptoms. PST was not only a reliable treatment approach to improving depression symptoms, but also it was superior to bupropion treatment to alleviate depression symptoms in women with infertility and SD. These results suggest that PST may be more effective compared with pharmacological therapy to treat depression symptoms in women with infertility and SD.

Consistent with this; study conducted by Faramarzi et al. (3) reported that group psychotherapy was superior to drug therapy in improving the well-being of women with infertility suffering from depression. In addition, they found that cognitive behavioral therapy was better to pharmacotherapy in relieving depression; depression symptoms were reduced to a greater extent than the control group. A review of literature suggests that counseling service is associated with lower depression, leading to higher life satisfaction compared to control group. Finally, the use of cognitive behavioral model can be effective in reducing frustration and depression, increasing skills, and improving the marital relationship and sexual satisfaction. Psychological model was an effective therapy for depression (35, 36).

Our findings showed that the decrease in the mean BDI score with BUP dosage of 150 mg per day was not statistically significant compared to the control group, although BUP group showed statistically significant improvement in depression symptom from beginning to end of research. Many trials showed that bupropion was efficacious in treatment MDD (27, 32).

It was found as an important antidepressant, and used to treat MDD (21). However, a few studies have observed no significant differences between bupropion and placebo groups on depression scores (19, 20) and bupropion 150 failed to demonstrate significant difference (19). It would be of great important to mention that low dose of bupropion was employed in subjects. Therefore, it seems that bupropion dosage of 150 mg once a day may have limited benefit for detecting difference between BUP and control groups in resolution of depression symptom. Furthermore, excluding the subjects with diagnosed MDD from this study may decrease the response rate to depression. Therefore, the different results of present research with others may relate to this model, especially in the infertility field. It should be noted that psychological placebo effects due to communication of the researcher with the patients in frequent assessments during the study should not be ignored. Further study need to distinguish factors leading to the lack of an effect.

## Conclusion

Psychosocial therapy was a superior treatment compared to bupropion for alleviating depression symptoms in women with infertility and SD. Therefore, counseling services and social support to recognize and treat depression and SD are necessary to establish in fertility centers.

There were a few limitations in this study. First, the data were collected from a small sample size of Iranian women with infertility; therefore, the findings cannot be generalized to all women with infertility or other populations and would require to be investigated in future research of a larger sample size. Another weakness of study was leak of follow-up. The strengths of this research include its use of a validated, self-reported Iranian version of the BDI. Also, for more effective treatment methods suggested that future studies consider the PST plus bupropion compared to each of them individually.
